# An Augmented Reality–Based Guide for Mechanical Ventilator Setup: Prospective Randomized Pilot Trial

**DOI:** 10.2196/38433

**Published:** 2022-07-22

**Authors:** Sejin Heo, Suhyeon Moon, Minha Kim, Minsu Park, Won Chul Cha, Meong Hi Son

**Affiliations:** 1 Department of Emergency Medicine Samsung Medical Center Sungkyunkwan University School of Medicine Seoul Republic of Korea; 2 Department of Digital Health Samsung Advanced Institute for Health Sciences and Technology Sungkyunkwan University Seoul Republic of Korea; 3 Research Institute for Future Medicine Samsung Medical Center Seoul Republic of Korea; 4 Department of Information and Statistics Chungnam National University Daejeon Republic of Korea; 5 Department of Pediatrics Samsung Medical Center Sungkyunkwan University School of Medicine Seoul Republic of Korea

**Keywords:** augmented reality, mechanical ventilation education, medical education, critical care, medical training, virtual reality, medical education, virtual education, nurse, nursing education, nursing, health care professional, learning platform, digital learning, digital health

## Abstract

**Background:**

Recently, the demand for mechanical ventilation (MV) has increased with the COVID-19 pandemic; however, the conventional approaches to MV training are resource intensive and require on-site training. Consequently, the need for independent learning platforms with remote assistance in institutions without resources has surged.

**Objective:**

This study aimed to determine the feasibility and effectiveness of an augmented reality (AR)–based self-learning platform for novices to set up a ventilator without on-site assistance.

**Methods:**

This prospective randomized controlled pilot study was conducted at Samsung Medical Center, Korea, from January to February 2022. Nurses with no prior experience of MV or AR were enrolled. We randomized the participants into 2 groups: manual and AR groups. Participants in the manual group used a printed manual and made a phone call for assistance, whereas participants in the AR group were guided by AR-based instructions and requested assistance with the head-mounted display. We compared the overall score of the procedure, required level of assistance, and user experience between the groups.

**Results:**

In total, 30 participants completed the entire procedure with or without remote assistance. Fewer participants requested assistance in the AR group compared to the manual group (7/15, 47.7% vs 14/15, 93.3%; *P*=.02). The number of steps that required assistance was also lower in the AR group compared to the manual group (n=13 vs n=33; *P*=.004). The AR group had a higher rating in predeveloped questions for confidence (median 3, IQR 2.50-4.00 vs median 2, IQR 2.00-3.00; *P*=.01), suitability of method (median 4, IQR 4.00-5.00 vs median 3, IQR 3.00-3.50; *P*=.01), and whether they intended to recommend AR systems to others (median 4, IQR 3.00-5.00 vs median 3, IQR 2.00-3.00; *P*=.002).

**Conclusions:**

AR-based instructions to set up a mechanical ventilator were feasible for novices who had no prior experience with MV or AR. Additionally, participants in the AR group required less assistance compared with those in the manual group, resulting in higher confidence after training.

**Trial Registration:**

ClinicalTrials.gov NCT05446896; https://beta.clinicaltrials.gov/study/NCT05446896

## Introduction

Mechanical ventilation (MV) is a lifesaving treatment that reduces the difficulty of breathing in patients and reverses acute life-threatening respiratory failure [1]. During the COVID-19 pandemic, the incidence of acute respiratory failure increased, leading to an increase in the demand for not only physical resources, such as ventilators and intensive care unit (ICU) beds, but also the ability to provide MV care expertise [2-4]. Effective and continuous MV education is important because adequate MV support improves clinical outcomes [5-8]. Regarding MV education, ICU nurses considered ventilator setup as an important topic; In ventilator setup, hands-on training is the most beneficial, suggesting that workshops or self-learning packages are not sufficient for novices to learn how to set up a ventilator [9]. However, conventional education usually focuses on theoretical knowledge (eg, prevention of infection and mode settings), and the type of hands-on training or bedside training that is required is human resource and time intensive, which limits educating several essential trainees [10-12].

Recently, augmented reality (AR) systems have been widely applied in medical education and training [13-16]. The AR system enables virtual objects to be overlaid onto a real-world environment by visualizing the physiological anatomy or enhancing the operator’s view [17,18]. A few AR-guided medical procedure training regimes have been reported in the emergency department and intensive care environments [13,15,19-21]. They suggested that AR systems are effective in step-by-step procedures; however, the studies were limited to procedures lasting less than 10 minutes or to simple steps that did not reflect the usual complexity of procedures performed in the ICU or emergency department [22,23]. Additionally, limited research has been conducted on the independence or accuracy of the step-by-step procedures in AR systems [19].

In this study, we aimed to determine the effectiveness and feasibility of AR-based learning for novices to set up a ventilator by focusing on independently completing the procedures and assessing the degree of assistance required. Additionally, we evaluated the step characteristics in terms of the precision and assistance required.

## Methods

This was a prospective randomized controlled pilot study conducted at Samsung Medical Center, Korea, from January to February 2022. We compared 2 modes of training, namely, the conventional method (via the printed manual) and the AR-based instructions. This study followed the CONSORT reporting guidelines (Multimedia Appendix 1).

### Ethical Considerations

The research design was approved by the institutional review board of Samsung Medical Center (2021-12-112). Prior to inclusion in the study, all participants provided written informed consent.

### Participants

We recruited nurses from the Samsung Medical Center who were interested in AR and ventilator education using a web-based hospital bulletin board. We enrolled nurses who had no prior experience with ventilator setup or AR systems, regardless of their work department or age. We excluded nurses who had already experienced setting up a ventilator or who had trouble wearing or using a head-mounted display (HMD). As this was a preliminary study, we were unable to determine the sample size. However, we referenced to past research on the step-by-step procedures with AR [19,24]. We set a target of 30 participants for recruitment.

### Study Design

Using a lottery method, we randomly assigned the participants to 2 groups. One group (the manual group) used a printed manual to set up a ventilator and the other group (the AR group) used AR-based instructions through an HMD—HoloLens 2 (Microsoft Corporation). The participants in the AR group were provided with 15 minutes of learning and practice time with HoloLens 2. If they needed assistance, participants in the manual group made a phone call, and those in the AR group requested it remotely with HoloLens 2; subsequently, both groups were assisted by the same ICU nurse. In the AR group, the participants shared the same view as the nurse using Microsoft Dynamics 365 Remote Assist, which allowed the ICU nurse to guide the participants through voice commands and drawing marks on their view. Both groups were surveyed immediately after the task.

### Instructions for Ventilator Setup

**Figure 1:**
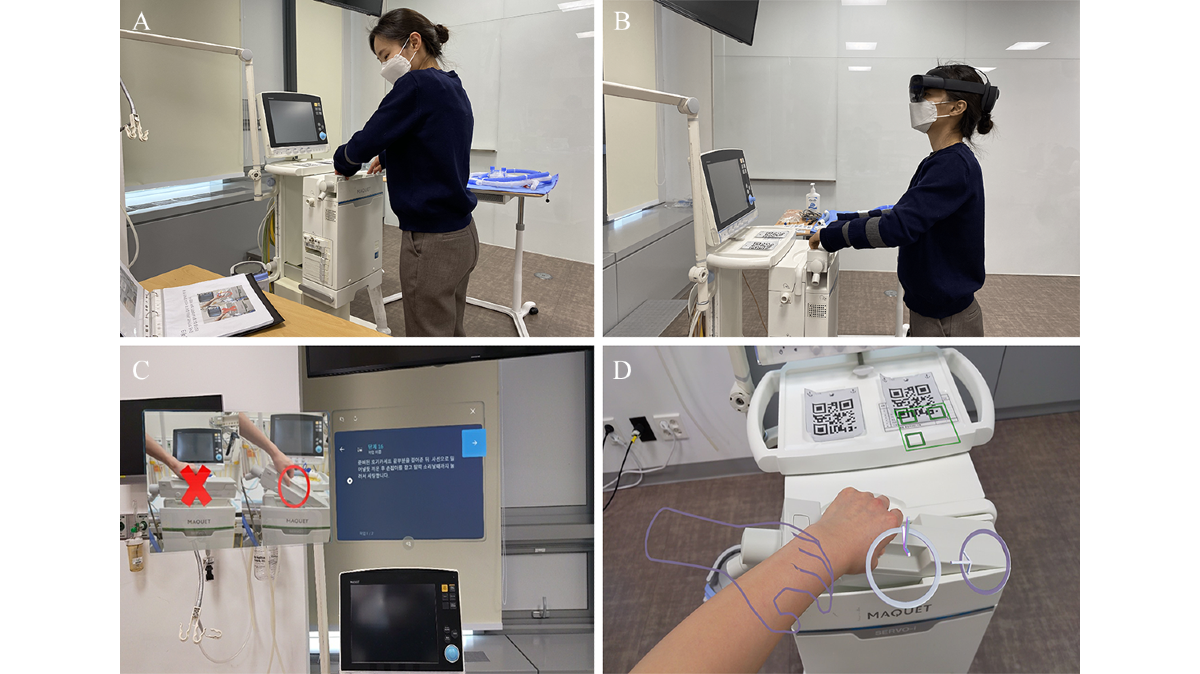
Scenes from the study: (A) trainee following the instructions with the printed manual; (B) trainee following the augmented reality (AR) instruction with head mounted display (HMD); (C) hologram instruction shown to trainee via HMD; (D) AR direction with hologram (3D object) overlaid on the mechanical ventilator.

The instructions to set up the Servo-i mechanical ventilator (Maquet) were developed by researchers, including emergency physicians, pediatricians, and ICU nurses. The instructions detailed the entire process, from plugging in a socket to turning on the power, by performing initial ventilator mode setting with 35 steps. The AR instructions were developed as a step-by-step guide with the same text and images as in the printed manual, using the Microsoft Dynamics 365 Guide. The AR instructions were delivered using Microsoft HoloLens 2. The device allows users to go back and forth through the entire procedure by gazing at the screen when required. Some steps had a guide with a hologram of the 3D objects to indicate the location of the steps and direct the action of the connecting parts (Figure 1).

### Evaluation of Outcomes

The primary outcome is the overall score of the procedure, which is a 100-point scale converted from the original score. Participants scored 1 point for each step if they successfully finished the step within 5 minutes and obtained a maximum score of 35. The secondary outcome was the required level of assistance (ie, the number of steps and the number of participants who required assistance, assistance frequency, and assistance time). We also evaluated the user’s experience with short questions on 3 themes: confidence, suitability, and whether they intended to recommend AR system to others [25,26]. All the participants were asked to respond to general questions on a 5-point scale ranging from 1 (strongly disagree) to 5 (strongly agree). The usability of the HMD in AR-based training was determined using previously validated system usability scale (SUS) standards [27,28].

### Statistical Analysis

All continuous variables are described as mean (SD) and median (IQR), and categorical variables are described as numbers and percentages. For continuous variables, we used the Wilcoxon rank-sum test; for categorical values, we used the chi-square test or Fisher exact test. A proportion test was performed to compare the proportions between the two groups. For all statistical analyses, *P*<.05 was considered statistically significant. The statistical analysis was performed using the R software (version 4.1.2; R Foundation for Statistical Computing).

## Results

### Characteristics of the Participants

A total of 31 nurses with no prior experience in setting up ventilators were enrolled, with the exception of 1 participant who had an HMD-related technical issue. The remaining 30 nurses completed the entire procedure and the surveys afterward. Each participant was randomly assigned to either the manual or the AR group. The participants ranged in age from 24-53 years and came from a variety of departments: 4 participants from outpatient nursing, 9 participants from inpatient nursing, 11 participants from specialized nursing, and 6 participants from administrative and educational departments. There were no significant differences in sex, age, work experience, prior observation experience, or department of work between the two groups (Table 1).

**Table 1:** Demographics of the study participants (N=30).

Characteristics		Manual group (n=15)	Augmented reality group (n=15)	*P* value
**Gender, n (%)**				.48
	Female	15 (100)	13 (86.7)	
	Male	0 (0)	2 (13.3)	
**Age (years), n (%)**				>.99
	20-30	9 (60)	8 (53.3)	
	30-40	3 (20)	4 (26.7)	
	≥40	3 (20)	3 (20)	
Age, median (Q1, Q3)		28 (26, 34.5)	28 (26.5, 35)	.84
**Work (years), n (%)**				.89
	<5	7 (46.7)	7 (46.7)	
	5-10	5 (33.3)	3 (20)	
	>10	3 (20)	5 (33.3)	
Previous experience of observing, n (%)		0 (0)	1 (6.7)	>.99
**Department of work, n (%)**				.84
	Outpatient nursing	3 (20)	1 (6.7)	
	Inpatient nursing	4 (26.7)	5 (33.3)	
	Specialized nursing^a^	5 (33.3)	6 (40)	
	Administration and educational part	3 (20)	3 (20)	

^a^In operating room, emergency department, radiology department, intensive care unit, and imaging center.

### Overall Performance

All 30 participants completed the entire procedure, with or without remote assistance. One participant in the AR group completed the procedure successfully without any assistance. Table 2 summarizes the overall results for the manual and AR groups. There was no significant difference in the overall score between the two groups, regardless of assistance. When only the steps without assistance were considered successfully passed, the median score was 88.57 (IQR 82.86-91.43) in the manual group and 91.43 (IQR 88.57-97.14; *P*=.10) in the AR group. However, if assisted steps were also considered as successful, the median score was 94.29 (IQR 91.43-94.29) in the manual group and 94.29 (IQR 92.86-97.14; *P*=.20) in the AR group.

The duration of the procedure between the two groups was not statistically significant. Without assistance, the median procedure time was 22.95 (IQR 19.37-24.69) minutes in the manual group and 23.95 (IQR 20.83-26.95; *P*=.60) minutes in the AR group. With assistance, the median procedure time was 25.32 (IQR 22.41-29.02) minutes in the manual group and 24.18 (IQR 22.37-28.41; *P*=.97) minutes in the AR group.

Multimedia Appendix 2 presents findings in terms of the step characteristics. We discovered that when following the directions of the ventilator or connecting and disconnecting materials such as a tube, circuit, or line, the manual group had a greater tendency to fail the steps or require assistance compared to the AR group. However, we did not identify any significant difference between the groups.

**Table 2:** Overall outcomes of augmented reality (AR)–based instructions (N=30).

Characteristics			Manual group (n=15)		AR group (n=15)		*P* value
**Score, median (IQR)**							
	without assistance	88.57 (82.86-91.43)		91.43 (88.57-97.14)		.10	
	with assistance	94.29 (91.43-94.29)		94.29 (92.86-97.14)		.20	
**Procedure time (min), median (IQR)**							
	without assistance	22.95 (19.37-24.69)		23.95 (20.83-26.95)		.60	
	with assistance	25.32 (22.41-29.02)		24.18 (22.37-28.41)		.97	
**Assistance**							
	Steps that needed assistance, n	33		13		.004	
	Steps that needed assistance per participant, median (IQR)	2 (1-2)		0 (0-1.5)		.03	
	Participants who requested assistance, n (%)	14 (93)		7 (48)		.02	
	Assistance time (min), median (IQR)	1.53 (0.78-2.98)		0 (0-3.02)		.12	

### Need of Assistance

The manual group required considerably more assistance than the AR group. The median number of steps that required assistance per participant was greater in the manual group compared to the AR group (median 2, IQR 1-2 vs median 0, IQR 0-1.5; *P*=.03; Table 2).

**Figure 2:**
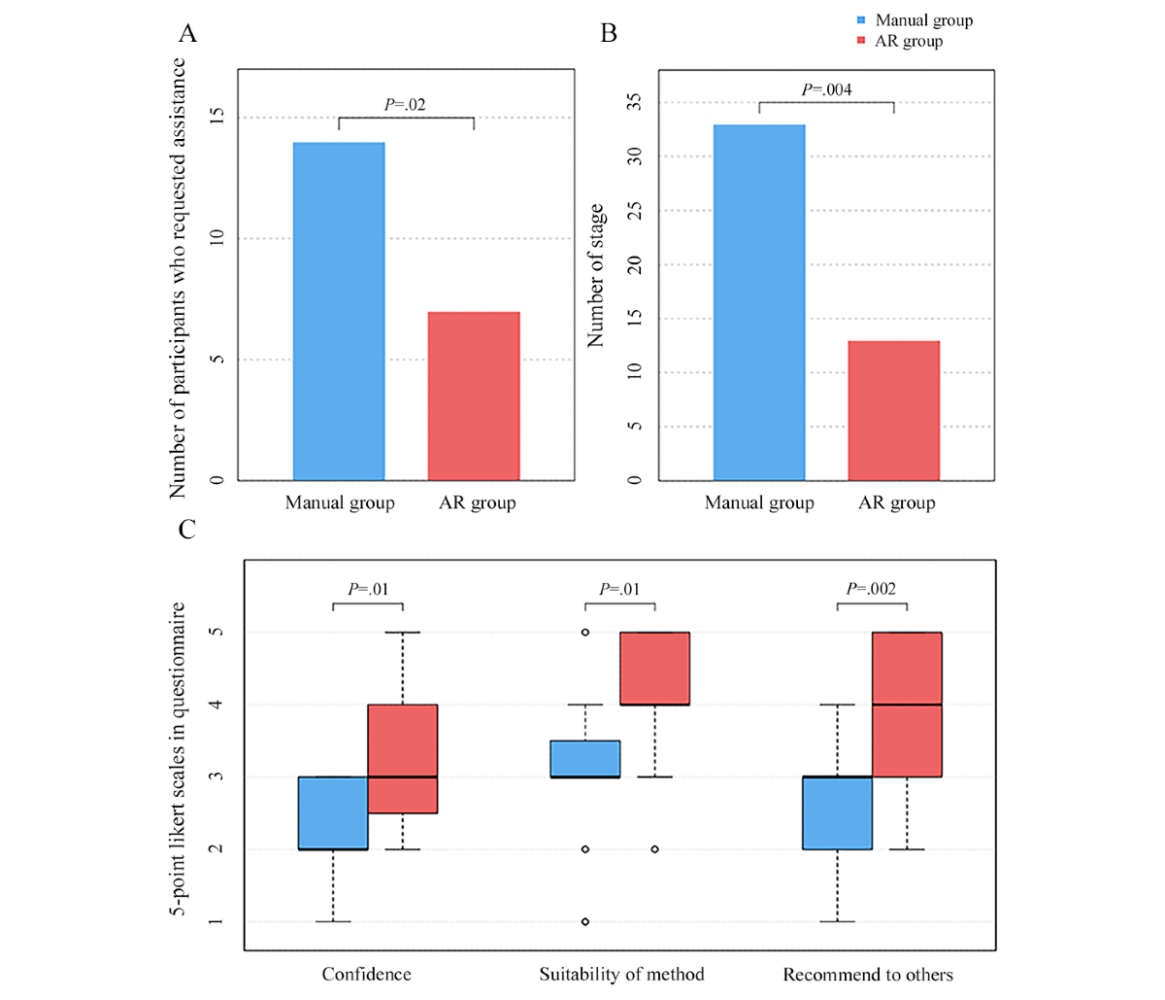
The effectiveness of augmented reality (AR)–based instruction for ventilator set up; (A) independent learning—the number of participants who requested assistance; (B) independent learning—number of stages that required assistance; (C) user experience.

The manual group had a greater proportion of participants who requested assistance compared to the AR group (14/15, 93.3% vs 7/15, 47.7%; *P*=.02; Figure 2A). Additionally, in the manual group, 33 requests for assistance were recorded, whereas only 13 requests were made in the AR group (*P*=.004; Figure 2B). There were no statistically significant differences in the time spent on assisting; the median was 1.53 (IQR 0.78-2.98) minutes for the manual group and 0 (IQR 0-3.02) minutes for the AR group (*P*=.12).

### Survey Outcomes

All the participants answered 3 general questions. Notably, only the AR group answered the SUS questions. Figure 2C shows the responses to the general questions on a 5-point Likert scale (from 1=strongly disagree to 5=strongly agree). The AR-based instructions received higher ratings for confidence (median 3, IQR 2.50-4.00 vs median 2, IQR 2.00-3.00; *P*=.01), suitability of method (median 4, IQR 4.00-5.00 vs median 3, IQR 3.00-3.50; *P*=.01), and whether they intended to recommend AR system to others (median 4, IQR 3.00-5.00 vs median 3, IQR 2.00-3.00; *P*=.002). The median of SUS score was 55 (IQR 47.5-67.5). Table 3 shows the details of each statement. Of all the statements, “well-integrated AR systems” received the best evaluation from the users, with the highest mean score and the lowest SD. Other statements such as “the simplicity of the system” (mean 3.7, SD 1.2), “ease of use” (mean 3.7, SD 1.1), and “technical assistance requirement” (mean 3.7, SD 1.1) obtained relatively high ratings.

**Table 3:** Evaluation of augmented reality (AR)–based instructions using the standardized system usability scale (SUS).

SUS questions	AR group (n=15), mean (SD)	AR group (n=15), median (IQR)
I think that I would like to use this system frequently.	3.5 (1.0)	4 (3.0-4.0)
I found the system unnecessarily complex.	3.7 (1.2)	4 (3.0-5.0)
I thought the system was easy to use.	3.7 (1.1)	4 (3.0-4.5)
I think that I would need the support of a technical person to be able to use this system.	3.7 (1.1)	4 (3.0-4.0)
I found that the various functions in this system were well integrated.	4.0 (0.6)	4 (3.0-5.0)
I thought there was too much inconsistency in this system.	1.9 (0.9)	2 (1.0-2.0)
I would imagine that most people would learn to use this system very quickly.	3.8 (1.0)	4 (3.0-5.0)
I found the system cumbersome to use.	2.4 (1.1)	2 (2.0-3.5)
I felt very confident using the system.	3.3 (1.0)	3 (2.5-4.0)
I needed to learn a lot of things before I could get going with this system.	3.3 (1.0)	4 (2.5-4.0)

## Discussion

### Principal Results

In this study, the participants had no prior experience with the ventilator setup or the HMD; additionally, all participants completed the entire procedure, from preparing materials to setting up the initial ventilator mode, prior to connecting to the patient. Moreover, the AR group was able to complete all the procedures following AR-based instructions in the planned design of the study, including a brief HMD practice and self-learning session. They required significantly less assistance compared to the manual group, and all assistance could be provided properly through a remote AR system. There were no technical issues or dropouts in either group.

Generally, hands-on training is required and beneficial when training trainees on complex procedures [29-31]. In addition, in a step-by-step procedure, failures in one step affect the subsequent steps, preventing the trainee from completing the process and requiring real-time guidance. However, experts in critical care cannot stay all day, and novices are required to use a systemic remote assistance when they face difficulties [32,33]. In addition, trainees do not have sufficient time to repeat the procedures, and when they practice alone, it is difficult to assist them in a proper manner.

AR-based training benefits both sides, as discussed above. The instructors are not required to be on-site, as the remote assistance software enables them to monitor and guide the trainee’s view as well as draw and transfer data, voice, and so on. Additionally, without prior technical knowledge or experience with computer programming, developing instructions for a procedure using HoloLens 2 software was possible in 2-3 hours. From the trainee’s perspective, they can learn frequently without visiting an education center or engaging in on-site instructions. Additionally, the desire to develop a contactless education platform has increased to protect health care workers and save on personal protection equipment [34].

The difference between the median number of assistance requests was not significant between the two groups; however, from a practical view, the difference in the workflow interruption between the two groups was more significant. A request for assistance resulted in procedure interruption by phone calls in the manual group and was difficult to support because they were held on to the phone. However, with an AR-based system, they could request help by speaking and connecting to the supervisor and be supported while continuing procedures.

### Comparison With Prior Work

Our results provide new evidence for the feasibility of AR-based independent learning in complex advanced procedures with 35 steps lasting more than 20 minutes. In earlier research, studies also discovered that participants were more satisfied with AR-based instructions than with conventional instructions [35]. They were more confident and felt that they had received adequate training for the procedure, and they intended to recommend AR systems to others. It is important for continuous learning to attain competency [36]. We expect that strong confidence and user satisfaction would result in greater willingness and self-practice for learning to set up a ventilator independently.

### Limitations

As a pilot study, there was no specific guideline regarding how to deal with technology issues, such as time for battery charging, overheating of the device without break time, and network instability. These issues were observed in a few cases but were solved without affecting the study; however, these issues will be addressed and planned in a larger-scale study.

Additionally, in the step-by-step procedures, the content of the errors is important; however, this was not addressed in this study. To extend AR-based training in other step-by-step advanced procedures and explore additional outcomes, considering the characteristics of steps and designing a training platform for suitable technology integration would be required.

### Conclusions

AR-based instructions to set up mechanical ventilator were feasible for novices with no prior experience with MV and AR. In addition, participants using AR required less assistance, resulting in higher confidence after training.

## Appendix

Multimedia Appendix 1 CONSORT-eHealth checklist (V 1.6.1).

Multimedia Appendix 2 Overall results of the study in a step-by-step manner.

## Abbreviations

AR: augmented reality
HMD: head-mounted display
ICU: intensive care unit
MV: mechanical ventilation
SUS: system usability scale
