# Supplementary material for: An Augmented Reality–Based Guide for Mechanical Ventilator Setup: Prospective Randomized Pilot Trial
Source: JMIR Serious Games. 2022 Jul 22;10(3):e38433. doi: 10.2196/38433 (PMC9356328; doi:10.2196/38433)
Supplement: Multimedia Appendix 1 [file games_v10i3e38433_app1.pdf]

CONSORT-EHEALTH Checklist V1.6.2 Report	Manuscript Number	38433

(based on CONSORT-EHEALTH V1.6), available at [http://tinyurl.com/consort-ehealth-v1-6].

Date completed

7/7/2022 13:09:44

by

Sejin Heo

An Augmented Reality-Based Guide for Mechanical Ventilator Setup: Prospective Randomized Pilot Trial

TITLE

1a-i) Identify the mode of delivery in the title

An "Augmented Reality-Based Guide" for Mechanical Ventilator Setup: Prospective Randomized Pilot Trial

1a-ii) Non-web-based components or important co-interventions in title

1a-iii) Primary condition or target group in the title

"An Augmented Reality-Based Guide for "Mechanical Ventilator Setup": Prospective Randomized Pilot Trial". This is for nursing education.

ABSTRACT

1b-i) Key features/functionalities/components of the intervention and comparator in the METHODS section of the ABSTRACT

This study aimed to determine the feasibility and effectiveness of an augmented reality (AR)-based self-learning platform for novices to set up a ventilator without on-site assistance. This was a prospective randomized controlled pilot study conducted at Samsung Medical Center, Korea from January to February 2022. Nurses with no prior experience of MV or AR were enrolled. "We randomized the participants into two groups: manual and AR groups. Participants in the manual group used a printed manual and made a phone call for assistance, whereas participants in the AR group were guided by AR-based instructions and requested assistance with the head-mounted device (HMD)." We compared the overall score of the procedure, required level of assistance, and user's experience between the groups.

1b-ii) Level of human involvement in the METHODS section of the ABSTRACT

1b-iii) Open vs. closed, web-based (self-assessment) vs. face-to-face assessments in the METHODS section of the ABSTRACT

1b-iv) RESULTS section in abstract must contain use data

1b-v) CONCLUSIONS/DISCUSSION in abstract for negative trials

INTRODUCTION

2a-i) Problem and the type of system/solution

During the COVID-19 pandemic, the incidence of acute respiratory failure increased, leading to an increase in the demand for not only physical resources such as ventilators and intensive care unit (ICU) beds, but also the ability to provide MV care expertise.

2a-ii) Scientific background, rationale: What is known about the (type of) system

Regarding MV education, ICU nurses responded to ventilator setup as an important topic, and for this, hands-on training is the most beneficial, suggesting that workshops or self-learning packages are not sufficient for novices to learn how to set up a ventilator. However, conventional education usually focuses on theoretical knowledge (prevention of infection and mode settings), and the type of hands-on training or bedside training that is required is human resource and time intensive, which limits educating several essential trainees.
Recently, augmented reality (AR) systems have been widely applied in medical education and training. The AR system enables virtual objects to be overlaid onto a real-world environment by visualizing the physiological anatomy or enhancing the operator's view. A few AR-guided medical procedure training regimes have been reported in the emergency department (ED) and intensive care environments.

Does your paper address CONSORT subitem 2b?

we aimed to determine the effectiveness and feasibility of AR-based learning for novices to set up a ventilator by focusing on independently completing the procedures and assessing the degree of assistance required. Additionally, we evaluated the step characteristics in terms of the precision and assistance required.

METHODS

3a) CONSORT: Description of trial design (such as parallel, factorial) including allocation ratio

This was a prospective randomized controlled pilot study conducted at Samsung Medical Center, Korea from January to February 2022.

3b) CONSORT: Important changes to methods after trial commencement (such as eligibility criteria), with reasons

We compared two modes of training, namely, the conventional method (printed manual) and AR-based instructions.

3b-i) Bug fixes, Downtimes, Content Changes

4a) CONSORT: Eligibility criteria for participants

We enrolled nurses who had no prior experience with ventilator setup or AR systems, regardless of their work department or age. We excluded nurses who had already experienced setting up a ventilator or who had trouble wearing or using a head-mounted device (HMD).

4a-i) Computer / Internet literacy

4a-ii) Open vs. closed, web-based vs. face-to-face assessments:

We recruited nurses from the Samsung Medical Center who were interested in AR and ventilator education using an online hospital bulletin board

4a-iii) Information giving during recruitment

4b) CONSORT: Settings and locations where the data were collected

This was a prospective randomized controlled pilot study conducted at Samsung Medical Center, Korea from January to February 2022.

4b-i) Report if outcomes were (self-)assessed through online questionnaires

Otherwise, we measured the time and score while participants conducted a setting of ventilator as primary outcome

4b-ii) Report how institutional affiliations are displayed

5) CONSORT: Describe the interventions for each group with sufficient details to allow replication, including how and when they were actually administered

5-i) Mention names, credential, affiliations of the developers, sponsors, and owners

5-ii) Describe the history/development process

5-iii) Revisions and updating

5-iv) Quality assurance methods

5-v) Ensure replicability by publishing the source code, and/or providing screenshots/screen-capture video, and/or providing flowcharts of the algorithms used

5-vi) Digital preservation

5-vii) Access

The participants in the AR group were provided 15 minutes of learning and practice time with the HoloLens 2. If they needed assistance, the participants in the manual group made a phone call and those in the AR group requested it remotely with HoloLens 2; subsequently both groups were assisted by the same ICU nurse. In the AR group, the participants shared the same view as the nurse utilizing the dynamic 365 remote assist (Software, Microsoft Corporation, Redmond, WA, USA), which allowed the ICU nurse to guide the participants through voice commands and by drawing marks on their view.

5-viii) Mode of delivery, features/functionalities/components of the intervention and comparator, and the theoretical framework

The instructions to set up the servo-i mechanical ventilator (Maquet, Rastatt, Germany) were developed by researchers, including emergency physicians, pediatricians, and ICU nurses. The instructions detailed the entire process, from plugging in a socket to turning on the power by performing initial ventilator mode setting with 35 steps. The AR instructions were developed as a step-by-step guide with the same text and images as in the printed manual, utilizing the dynamic 365 Guide (Software, Microsoft Corporation, Redmond, WA, USA). The AR instructions were delivered using the Microsoft HoloLens 2. The device allows users to go back and forth through the entire procedure by gazing at the screen when required. Some steps had a guide with a hologram of the 3D objects to indicate the location of the steps and direct the action of the connecting parts.

**5-ix) Describe use parameters**		
**5-x) Clarify the level of human involvement**		
**5-xi) Report any prompts/reminders used**		
The study was regarding to nursing education, subitem 5-xi was not indicated.		
**5-xii) Describe any co-interventions (incl. training/support)**		
In this study, only Hololens 2 was used for intervention, so the other co-intervention was not existed.		
**6a) CONSORT: Completely defined pre-specified primary and secondary outcome measures, including how and when they were assessed**		
The primary outcome is the overall score of the procedure, which is a 100-point scale converted from the original score. The participants scored 1 point for each step if they successfully finished the step within five minutes and obtained a maximum score of 35. The secondary outcome was the required level of assistance (number of steps and participants, assistance frequency, and assistance time). We also evaluated the user's experience with short questions on three themes: confidence, suitability, and whether they intended to recommend to others. All the participants were asked to respond to general questions on a five-point scale ranging from 1 (strongly disagree) to 5 (strongly agree). The usability of the HMD in AR-based training was determined using previously validated system usability scale (SUS) standards.		
**6a-i) Online questionnaires: describe if they were validated for online use and apply CHERRIES items to describe how the questionnaires were designed/deployed**		
**6a-ii) Describe whether and how "use" (including intensity of use/dosage) was defined/measured/monitored**		
**6a-iii) Describe whether, how, and when qualitative feedback from participants was obtained**		
**6b) CONSORT: Any changes to trial outcomes after the trial commenced, with reasons**		
This was a prospective randomized controlled pilot study conducted at Samsung Medical Center, Korea from January to February 2022.		
**7a) CONSORT: How sample size was determined**		
**7a-i) Describe whether and how expected attrition was taken into account when calculating the sample size**		
**7b) CONSORT: When applicable, explanation of any interim analyses and stopping guidelines**		
The primary outcome is the overall score of the procedure, which is a 100-point scale converted from the original score. The participants scored 1 point for each step if they successfully finished the step within five minutes and obtained a maximum score of 35. The secondary outcome was the required level of assistance (number of steps and participants, assistance frequency, and assistance time). We also evaluated the user's experience with short questions on three themes: confidence, suitability, and whether they intended to recommend to others. All the participants were asked to respond to general questions on a five-point scale ranging from 1 (strongly disagree) to 5 (strongly agree). The usability of the HMD in AR-based training was determined using previously validated system usability scale (SUS) standards.		
**8a) CONSORT: Method used to generate the random allocation sequence**		
The participants were randomly assigned to two groups using a lottery method.		
**8b) CONSORT: Type of randomisation; details of any restriction (such as blocking and block size)**		
The participants were randomly assigned to two groups using a lottery method. One group (manual group) used a printed manual to set up a ventilator and the other group (AR group) used AR-based instructions through a HMD HoloLens 2 (Microsoft Corporation, Redmond, WA, USA).		
**9) CONSORT: Mechanism used to implement the random allocation sequence (such as sequentially numbered containers), describing any steps taken to conceal the sequence until interventions were assigned**		
The participants were randomly assigned to two groups using a lottery method.		
**10) CONSORT: Who generated the random allocation sequence, who enrolled participants, and who assigned participants to interventions**		
KM-M generated a draw, H-SJ enrolled participants, and we assigned participants as a results of lottery method.		
**11a) CONSORT: Blinding - If done, who was blinded after assignment to interventions (for example, participants, care providers, those assessing outcomes) and how**		
**11a-i) Specify who was blinded, and who wasn't**		
Nothing of them was blinded.		
**11a-ii) Discuss e.g., whether participants knew which intervention was the "intervention of interest" and which one was the "comparator"**		
**11b) CONSORT: If relevant, description of the similarity of interventions**		
the item is not applicable/relevant for our study		
**12a) CONSORT: Statistical methods used to compare groups for primary and secondary outcomes**		
All the continuous variables are described as mean ± SD and median (IQR) and categorical variables as n (%). For continuous variables, we used the Wilcoxon rank-sum test; for categorical values, we used the chi-square test or Fisher's exact test. A proportion test was performed to compare the proportions between the two groups. For all statistical analyses, a P-value<.05 was considered as statistically significant. The statistical analysis was performed using the R software (version 4.1.2, R Foundation for Statistical Computing, Vienna, Austria).		
**12a-i) Imputation techniques to deal with attrition / missing values**		
The item is not applicable/relevant for our study		
**12b) CONSORT: Methods for additional analyses, such as subgroup analyses and adjusted analyses**		
We did not conduct additional analyses		
RESULTS		
**13a) CONSORT: For each group, the numbers of participants who were randomly assigned, received intended treatment, and were analysed for the primary outcome**		
All 30 participants completed the entire procedure, with or without remote assistance		
**13b) CONSORT: For each group, losses and exclusions after randomisation, together with reasons**		
A total of 31 nurses with no prior experience in setting up ventilators were enrolled		
**13b-i) Attrition diagram**		
**14a) CONSORT: Dates defining the periods of recruitment and follow-up**		
the item is not applicable/relevant for our study: because the study objective is nursing education		
**14a-i) Indicate if critical "secular events" fell into the study period**		
**14b) CONSORT: Why the trial ended or was stopped (early)**		
the item is not applicable/relevant for our study: because the study objective is nursing education		
**15) CONSORT: A table showing baseline demographic and clinical characteristics for each group**		
We described the participant's demographics in Table 1		
**15-i) Report demographics associated with digital divide issues**		
We described the participant's demographics in Table 1		
**16a) CONSORT: For each group, number of participants (denominator) included in each analysis and whether the analysis was by original assigned groups**		
**16-i) Report multiple "denominators" and provide definitions**		
the item is not applicable/relevant for our study: because the study objective is nursing education		
**16-ii) Primary analysis should be intent-to-treat**		
**17a) CONSORT: For each primary and secondary outcome, results for each group, and the estimated effect size and its precision (such as 95% confidence interval)**		
We described the overall outcomes in Table 2.		
**17a-i) Presentation of process outcomes such as metrics of use and intensity of use**		
**17b) CONSORT: For binary outcomes, presentation of both absolute and relative effect sizes is recommended**		
We described the overall outcomes in Table 2.		
**18) CONSORT: Results of any other analyses performed, including subgroup analyses and adjusted analyses, distinguishing pre-specified from exploratory**		
the item is not applicable/relevant for out study. we did not any other subgroup analyses.		
**18-i) Subgroup analysis of comparing only users**		
**19) CONSORT: All important harms or unintended effects in each group**		

The item is not applicable/relevant for our study because the study objective was nursing education		
**19-i) Include privacy breaches, technical problems**		
**19-ii) Include qualitative feedback from participants or observations from staff/researchers**		
DISCUSSION		
**20) CONSORT: Trial limitations, addressing sources of potential bias, imprecision, multiplicity of analyses**		
**20-i) Typical limitations in ehealth trials**		
As a pilot study, there was no specific guideline regarding how to deal with technology issues, such as time for battery charging, overheating of the device without break time, and network instability. These issues were observed in a few cases but were solved without affecting the study; however, these issues will be addressed and planned in a larger scale study.<br>Additionally, in the step-by-step procedures, the content of the errors is important; however, this was not addressed in this study. To extend AR-based training in other step-by-step advanced procedures and explore additional outcomes, considering the characteristics of steps and designing a training platform for suitable technology integration would be required.		
**21) CONSORT: Generalisability (external validity, applicability) of the trial findings**		
**21-i) Generalizability to other populations**		
**21-ii) Discuss if there were elements in the RCT that would be different in a routine application setting**		
**22) CONSORT: Interpretation consistent with results, balancing benefits and harms, and considering other relevant evidence**		
**22-i) Restate study questions and summarize the answers suggested by the data, starting with primary outcomes and process outcomes (use)**		
In this study, the participants had no prior experience with the ventilator setup or AR HMD; additionally, all novices completed the entire procedure, from preparing materials to setting up the initial ventilator mode prior to connecting to the patient. Moreover, the AR group was able to complete all the procedures following AR-based instructions in the planned design of the study, including a brief AR HMD practice and self-learning session. They required significantly lesser assistance than the manual group, and all assistance could be provided properly through remote AR systems. There were no technical issues or dropouts in either group.		
**22-ii) Highlight unanswered new questions, suggest future research**		
Other information		
**23) CONSORT: Registration number and name of trial registry**		
ClinicalTrials.gov ID : NCT05446896		
**24) CONSORT: Where the full trial protocol can be accessed, if available**		
We registered the study at ClinicalTrials.gov		
**25) CONSORT: Sources of funding and other support (such as supply of drugs), role of funders**		
This study was not funded.		
**X26-i) Comment on ethics committee approval**		
**x26-ii) Outline informed consent procedures**		
**X26-iii) Safety and security procedures**		
**X27-i) State the relation of the study team towards the system being evaluated**		
