# Supplementary material for: An Augmented Reality–Based Guide for Mechanical Ventilator Setup: Prospective Randomized Pilot Trial
Source: JMIR Serious Games. 2022 Jul 22;10(3):e38433. doi: 10.2196/38433 (PMC9356328; doi:10.2196/38433)
Supplement: Multimedia Appendix 2 [file games_v10i3e38433_app2.docx]

**Multimedia Appendix 2.** Overall results of the study in a step-by-step manner

| **Grouped step description** | **Step description** | **Step number** | **Incorrect or needed assistance to pass,**  **n (%)** | |  |
| --- | --- | --- | --- | --- | --- |
|  |  |  | **Manual group** | **AR group** | |
| **Preparation materials** | Preparing materials | 1 | 1 (6.7) | 0 (0) | |
| **Expiratory cassette setting** | Opening the ventilator body | 2 | 1 (6.7) | 1 (6.7) | |
|  | Connecting the exhalation cassette | 3 | 9 (60) | 6 (40) | |
|  | Closing the ventilator body | 4 | 0 (0) | 0 (0) | |
| **Starting pre-use check** | Starting initial step of pre use check | 5 | 0 (0) | 0 (0) | |
|  | Connecting the test tube | 6 | 11 (73.3) | 6 (40) | |
|  | pre use check | 7 | 8 (53.3) | 3 (20) | |
|  | Testing the plug in | 8 | 3 (20) | 1 (6.7) | |
|  | Removal of test tube | 9 | 4 (26.7) | 1 (6.7) | |
| **Set up a ventilator humidifier** | Preparing humidifier | 10 | 1 (6.7) | 0 (0) | |
|  | Connecting to humidifier ventilator | 11 | 1 (6.7) | 0 (0) | |
|  | Connecting a distilled water | 12 | 0 (0) | 0 (0) | |
|  | Checking connection of humidifier and distilled water | 13 | 0 (0) | 0 (0) | |
| **Breathing circuit settings** | Connecting the short tube | 14 | 6 (40) | 3 (20) | |
|  | Connecting the blue circuit | 15 | 1 (6.7) | 0 (0) | |
|  | Connecting the white circuit | 16 | 5 (33.3) | 3 (20) | |
|  | Connecting the circuit support arm | 17 | 1 (6.7) | 0 (0) | |
| **Cable setting for heat transfer** | Preparing a heat cable | 18 | 0 (0) | 0 (0) | |
|  | Connecting a yellow code | 19 | 2 (13.3) | 5 (33.3) | |
|  | Connecting a three-piece cable | 20 | 3 (20) | 1 (6.7) | |
|  | Connecting a two-piece cable | 21 | 1 (6.7) | 0 (0) | |
| **Cable setting for temperature control** | Preparing the temperature measuring cable | 22 | 0 (0) | 0 (0) | |
|  | Connecting a blue code | 23 | 2 (13.3) | 1 (6.7) | |
|  | Connecting the probe | 24 | 3 (20) | 4 (26.7) | |
|  | Connecting the airway probe | 25 | 1 (6.7) | 2 (13.3) | |
| **Completing pre use check** | Pop-up window checking for Circuit and y-piece connection | 26 | 2 (13.3) | 0 (0) | |
|  | Checking of circuit compliance | 27 | 0 (0) | 4 (26.7) | |
|  | Confirming the result of the pre use check | 28 | 0 (0) | 0 (0) | |
|  | Deleting data | 29 | 0 (0) | 0 (0) | |
|  | Completing pre use check and ventilator mode setting | 30 | 0 (0) | 0 (0) | |
| **Ventilator mode set up** | Starting ventilator mode setting | 31 | 0 (0) | 0 (0) | |
|  | Selection of ventilator mode 1 | 32 | 1 (6.7) | 0 (0) | |
|  | Selection of ventilator mode 2 | 33 | 1 (6.7) | 0 (0) | |
|  | Adjusting ventilator setting values | 34 | 1 (6.7) | 0 (0) | |
|  | Start ventilation | 35 | 0 (0) | 0 (0) | |

|  | A step where 3D hologram guide appears |  |  |  |  |  |
| --- | --- | --- | --- | --- | --- | --- |

This is a Multimedia Appendix to a full manuscript published in the J Med Internet Res. For full copyright and citation information see http://dx.doi.org/10.2196/jmir.xxxx
